# Survey and analysis on the resource situation of primary health care institutions in rural China

**DOI:** 10.3389/fpubh.2024.1394527

**Published:** 2024-06-11

**Authors:** Zhaoting Zhang

**Affiliations:** School of Public Policy and Management, China University of Mining and Technology, Xuzhou, China

**Keywords:** rural towns, medical institutions, health resources, medical services, national average

## Abstract

**Background:**

China’s rural population is immense, and to ensure the well-being of rural residents through healthcare services, it is essential to analyze the resources of rural grassroots healthcare institutions in China. The objective is to examine the discrepancies and deficiencies in resources between rural grassroots healthcare institutions and the national average, providing a basis for future improvements and supplementation of rural healthcare resources.

**Methodology:**

The study analyzed data from 2020 to 2022 on the number of healthcare establishments, the capacity of hospital beds, the number of healthcare professionals, and the number of physicians in both rural and national settings. Additionally, it examined the medical service conditions and ratios of township health centers in rural areas to assess the resource gap between rural areas and the national average.

**Results:**

Healthcare establishments: On average, there were 2.2 fewer healthcare institutions per 10,000 persons in rural areas compared to the national average over three years. Hospital beds: On average, there were approximately 36 fewer hospital beds per 10,000 persons in rural areas compared to the national average over three years. Healthcare professionals and physicians: On average, there were about 48 fewer healthcare technical personnel and 10 fewer practicing (including assistant) physicians per 10,000 persons in rural areas compared to the national average over three years.

**Conclusion:**

Compared to the national average, there are significant discrepancies and deficiencies in grassroots healthcare resources in rural China. This underscores the necessity of increasing funding to progressively enhance the number of healthcare institutions in rural areas, expand the number of healthcare personnel, and elevate medical standards to better align with national benchmarks. Improving rural healthcare resources will strategically equip these institutions to cater to rural communities and effectively handle public health emergencies. Ensuring that the rural population in China has equal access to healthcare services as the rest of the country is crucial for promoting the well-being of rural residents and achieving health equity.

## 1 Introduction

There are large populations spread out over large areas in rural China. With 491.04 million people, or 34.78% of the entire population, residing in rural areas of China as of the end of 2022 (1). There are more than 691,500 administrative villages and 38,602 township-level administrative regions that make up the administrative backbone of rural governance (2). When it comes to managing and providing services to rural areas around the country, this vast administrative network is crucial. But even with all this infrastructure, rural communities still have a lot of problems, especially when it comes to healthcare (3). The COVID-19 pandemic, which broke out in late 2019, revealed gaps in rural public health interventions and worsened preexisting imbalances (4, 5). There has to be better healthcare infrastructure because the virus was migratory and spread throughout rural areas, putting a burden on medical institutions and resources (4, 6).

There is an immediate need to improve healthcare infrastructure in rural areas due to the changing nature of public health crises. New infectious illnesses and public health emergencies continue to be major concerns, even if the COVID-19 pandemic has highlighted the weaknesses of healthcare in rural areas (7). Regions characterized by high unemployment rates and a lack of health insurance coverage show limited availability of COVID-19 testing and immunization facilities compared to wealthier areas (8). In recent years, the “Healthy China 2030 Strategy” has underscored the importance of health in China, a focus further intensified by the COVID-19 pandemic in 2020 (9), and people started living with anxiety and depression (10). Health issues in rural areas are complicated and unpredictable, made worse by factors including population growth, environmental deterioration, and climate change. To successfully address both the urgent healthcare demands and the long-term capacity to minimize future health crises, it is necessary to enhance the resilience and responsiveness of rural healthcare (11). To help rural healthcare in China progress, this study aims to delve into these complexities and look at how socio-economic issues intersect with healthcare services. The goal is to provide detailed insights and practical recommendations (5).

The safety of people living in rural areas depends on filling this gap. Healthcare for rural people has long been a priority for the World Health Organization, which has pushed for countries to invest in training and maintaining health workers in outlying regions (12). A care pathway is a means to enhance the quality of care across the continuum by improving risk-adjusted patient outcomes, promoting patient safety, increasing patient satisfaction, and optimizing the use of resources (13). To attain healthcare parity between urban and rural communities, academics in China continue to support the call for improved rural medical services (14). The healthcare insurance system is a significant institutional arrangement aimed at safeguarding the health of residents, enhancing their well-being, and maintaining social harmony and stability (15). Similarly, Patients with other diseases like diabetes in rural areas have poor glycemic control and a high incidence of diabetic complications. Patients with diabetes in rural areas have poor knowledge and inadequate health information-seeking behavior (16).

The purpose of this research is to assess the sufficiency of rural Chinese healthcare infrastructure, with an eye on how well it can address the medical service requirements of rural inhabitants. This research seeks to enhance rural public health and medical infrastructure by analyzing the current state of rural health management institutions through a statistical survey. The goal is to identify areas that could be improved and to influence future efforts in this area. To accomplish this, the study will examine a range of indicators, such as the quantity and quality of healthcare facilities and beds, the accessibility of healthcare workers, and the standard of medical treatment provided at the community level. The purpose of the present investigation is to evaluate the efficiency of current healthcare resources in meeting the health demands of rural areas by comparing the results to national averages and standards.

To lay the groundwork for creating individualized interventions, these investigations will shed light on the amount, condition, and degree of uniformity of rural healthcare services. This project seeks to improve the ability of local healthcare institutions in rural areas to respond to public health issues and meet the health security demands of local citizens by providing them with data-driven solutions. Recognizing the larger socio-economic dynamics affecting rural healthcare in China is vital for further explaining the significance of this study (17). Factors such as physical distance, inadequate infrastructure, and discrepancies in resource distribution frequently make it difficult for rural communities to get appropriate healthcare, compared to their metropolitan counterparts. An aging population in rural areas with unique healthcare requirements compounds these problems as younger people leave for cities in search of better prospects. Furthermore, the demand for healthcare resources and services may be shaped by the traditional beliefs and practices of healthcare that are common in rural areas. These practices and beliefs may impact how people seek medical treatment and how they perceive modern medical services.

## 2 Theoretical background

This study on primary healthcare resource situations in rural China is based on a complex theoretical framework. The study is positioned within frameworks that highlight the significance of primary healthcare in promoting population health and the relevance of equal access to healthcare services, drawing from literature on healthcare policy and management (18, 19). Comprehensive, community-based healthcare practices are particularly important in rural areas, as highlighted by the Alma-Ata Declaration’s principles of primary healthcare (20). Study reveals that a significant portion of Egyptian private hospitals exhibit inefficiencies, particularly in technical efficiency (21). Numbers of factors play crucial roles in influencing both operational and financial efficiency, highlighting areas for improvement to enhance overall performance in the healthcare sector.

Further, the research is in line with the WHO’s Health Systems Framework, which stresses the importance of healthcare facilities, personnel, and service provision in attaining healthcare coverage for all (22). Research on rural health disparities also helps put the specific problems, such as a lack of funding, qualified medical professionals, and physical space, that rural healthcare systems face into perspective (23). This study intends to promote rural healthcare and address gaps in access and quality of care in China by integrating various theoretical views and contributing to the growth of evidence-based policies (24).

## 3 Literature review

Persistent difficulties and inequalities in healthcare access and provision are highlighted in the research related to primary healthcare institutions in rural China (25, 26). Inadequate infrastructure, healthcare worker shortages, and resource inequality between urban and rural areas have been identified in earlier research (27, 28). Healthcare utilization and health outcomes in rural communities are influenced by socioeconomic factors, such as poverty and education levels, according to a study (19, 29).

Rural healthcare systems are more susceptible to public health crises like the COVID-19 pandemic, and studies looking at how these systems were handled have highlighted the importance of being better prepared and adaptable (30). Research has also looked at policy initiatives and community-based approaches as potential ways to improve healthcare delivery in rural areas (31, 32). This literature review (Table 1) lays the framework for the current survey and analysis to help address these critical healthcare challenges by combining these findings and providing a comprehensive understanding of the socioeconomic variables that influence the resource status of primary healthcare institutions in rural China.

**Table 1:** List of relevant publications on the status of primary healthcare in chronological order.

Reported year	Description
2010	Title: Increasing Access to Health Workers in Remote and Rural Areas through Improved Retention: Global Policy Recommendations.
	Authors: World Health Organization
	Key Findings: Highlighted the significance of resolving healthcare inequalities in rural areas by focusing on the needs of those living there.
2010	Title: Evaluated Strategies to Increase Attraction and Retention of Health Workers in Remote and Rural Areas.
	Authors: C. Dolea, L. Stormont
	Key Findings: Addressed issues including workload and professional development possibilities as we looked for ways to keep healthcare workers in remote areas.
2013	Title: Enhancing Staffing in Rural Community Health Centers Can Help Improve Behavioral Health Care.
	Authors: X. Han, L. Ku
	Key Findings: Highlighted the potential of community health centers to increase access to healthcare by studying their efficacy in providing primary healthcare services in rural areas.
2015	Title: Consolidating the Social Health Insurance Schemes in China: Toward an Equitable and Efficient Health System.
	Authors: Q. Meng, et al.
	Key Findings: Results showed that rural areas lacked access to healthcare compared to urban areas, demonstrating the necessity for governmental initiatives to address this issue.
2018	Title: Health Status in a Transitional Society: Urban–Rural Disparities from a Dynamic Perspective in China.
	Authors: J. Jiang, P. Wang
	Key Findings: Examined difficulties such as resource limitations and uneven distribution of healthcare facilities, providing ways for increasing rural healthcare equity.
2020	Title: Impacts of COVID-19 on Agriculture and Rural Poverty in China.
	Authors: J. Huang
	Key Findings: Addressed the ways in which the pandemic affected healthcare systems in rural areas, finding weak spots and highlighting the critical need to fortify these systems immediately.
2022	Title: Resource Allocation Equity in China’s Rural Three-Tier Healthcare System.
	Authors: Y. Ao, et al.
	Key Findings: Examination of the challenges of providing healthcare in rural areas and found ways to enhance the distribution of resources and the quality of services offered.
2022	Title: Traditional Chinese Medicine to Improve Rural Health in South Africa: A Case Study for Gauteng.
	Authors: Z. Hu, R. Venketsamy
	Key Findings: Investigated the pros and cons of incorporating traditional medical practices into healthcare systems in rural areas.
2023	Title: Impact of Health Insurance Equity on Poverty Vulnerability: Evidence from Urban–Rural Health Insurance Integration in Rural China.
	Authors: Z. Li, Y. Chen, J. Ding
	Key Findings: Researchers looked into how health insurance plans affect healthcare use in rural areas and what variables impact people’s ability to get the treatment they need.
2024	Title: The Future of Healthcare and Patient-Centric Care: Digital Innovations, Trends, and Predictions.
	Authors: S. Aminabee
	Key Findings: Highlighted prospects for utilizing digital solutions after researching the significance of technological advancements in enhancing healthcare access and quality in rural areas.

## 4 Materials and methods

In China, rural primary medical establishments principally comprise township health clinics, village health centers, rural community health service centers, and others (12). The construction and improvement of these institutions aim to enhance the basic medical service levels of rural healthcare facilities, ensuring that rural residents can access timely and effective medical services. The development and quantity of rural grassroots medical institutions and resources are influenced by various factors, including policies, socio-economic development, changes in population structure, and advancements in medical technology (33). To determine if grassroots medical resources in rural regions are sufficient to meet their development needs, it is necessary to evaluate several indicators and determinants. These include indicators related to physical facilities and medical services. This study aimed to compare healthcare facility resources in rural and national contexts using the following variables: number of healthcare facilities, capacity of hospital beds, healthcare technical workers, and physicians. The goal was to draw comparisons with the national average. All of these things put the state of rural grassroots healthcare facilities in China into context and show their current level of development (5). Factors like changes in the national and rural population structure are also taken into account in the analysis.

### 4.1 Survey on the number of healthcare institutions and hospital bed capacity

The quantity of healthcare institutions and the number of hospital beds are crucial indicators for assessing the richness of medical resources in a region. They have a significant impact on the accessibility of medical services for residents and the coverage of medical services. The number of healthcare institutions in rural areas reflects the accessibility of the coverage of medical services (34). Rural populations would have easier access to basic medical services, medical services would be less overwhelmed, and healthcare institutions would have more options if there were more of them. A hospital’s ability to treat patients is proportional to the number of beds it has. Better preparedness for emergencies, such as infectious disease epidemics, and enhanced skills for disease prevention and control are both contributed by an increase in bed capacity. The term “bed capacity” is used in this study to describe the total number of permanent hospital beds at the end of the year. This number includes both ordinary and basic beds, as well as intensive care beds, beds that are currently being cleaned and repaired, and beds that are temporarily unavailable because of renovations or expansions. The following types of beds are not included: maternity, newborn, delivery room, reserved, observation, temporary extra, and family member beds (35). When evaluating the number of healthcare institutions and hospital bed capacity in rural areas, demonstrating shifts within the rural population must be taken into consideration. According to the data from the seventh national population census, the rural population residing in mainland China was 509.78 million people in 2020, representing for 36.11% of the national population proportion (36). In 2021, according to the statistics from the China National Bureau of Statistics, the permanent rural population was 498.35 million people, constituting 35.28% of the national population (37). By the end of 2022, the China National Bureau of Statistics reported that the permanent rural population was 491.04 million people, making up 34.78% of the national population (1). The total mainland population in China was 1,411.75 million people (excluding Hong Kong, Macau, and Taiwan) (1).

As of the end of 2022, rural areas housed 33,917 township health clinics and 587,749 village health centers in rural areas. Compared to 2021, this represents a decrease of 1,026 and 11,543, respectively. In comparison to 2020, the numbers decreased by 1,845 and 21,079, respectively. Correspondingly, the total number of medical and health institutions nationwide at the end of 2022 was 1,032,918. This marks an increase of 1,983 from the previous year and 9,996 from 2020. The aggregate comprises 36,976 hospitals and 979,768 grassroots medical and health institutions (including community-based institutions and rural health centers). Compared to 2021, hospitals increased by 406, and grassroots medical and health institutions increased by 1,978. In comparison to 2020, hospitals increased by 1,582, and grassroots medical and health institutions increased by 9,732. In terms of bed capacity, as of the end of 2022, rural township health clinics had a total of 1,455,876 beds. This represents an increase of 38,466 beds from 2021 and 65,551 beds from 2020. Nationwide, the total number of beds in medical and health institutions was 9.75 million. Compared to 2021, this is an increase of 299,800 beds, and compared to 2020, it is an increase of 649,200 beds. This total includes 7.663 million beds in hospitals and 1.744 million beds in grassroots medical and health institutions. Compared to 2021, hospital beds increased by 248,700, and grassroots medical and health institution beds increased by 44,600. There was a 531,700-bed increase in hospitals and a 95,000-bed increase in community health centers and other grassroots medical facilities compared to 2020 (38–40). Although the number of rural township health clinics has decreased, the data shows that their bed capacity is on the rise. Table 2 is a statistical table of changes in the number of beds in national and rural health and medical institutions and medical institutions in 3 years from 2020 to 2022. The table counts the increases and decreases in various medical institutions in the past 3 years.

**Table 2:** Statistical table of national medical and health institutions and number of beds in 2020–2021.

Number of medical and health institutions and number of beds						
Institutions category	Number of institutions (number)			Number of beds (beds)		
	2020	2021	2022	2020	2021	2022
1. Hospital	35,394	36,570	36,976	7,131,186	7,414,228	7,662,929
Public hospital	11,870	11,804	11,746	5,090,558	5,207,727	5,363,364
Private hospital	23,524	24,766	25,230	2,040,628	2,206,501	2,299,565
Among them: tertiary hospitals	2,996	3,275	3,523	3,002,503	3,230,629	3,445,405
Secondary hospital	10,404	10,848	11,145	2,718,116	2,743,079	2,773,482
First class hospital	12,252	12,649	12,815	712,732	726,054	732,490
2. Professional public health institutions	14,492	13,276	12,436	296,063	301,566	313,558
Among them: Centers for Disease Control and Prevention	3,384	3,376	3,386		-	
Specialized disease prevention and treatment institutions	1,048	932	856	42,323	40,611	39,133
Maternal and child health care institutions, etc.	3,052	3,032	3,031	253,740	260,955	274,425
Health Supervision Institute (Center)	2,934	3,010	2,944			
Family planning technical service agencies, etc.	4,074	2,926	2,219			
3. Community primary medical and health institutions	325,446	343,555	358,102	259,059	282,366	288,549
Among them: community health service center	9,826	10,122	10,353	225,539	239,139	251,453
Community health service station	25,539	26,038	26,095	12,804	12,581	11,601
Clinic (nursing station), etc.	290,081	307,395	321,654	20,716	30,646	25,495
4. Rural Health Center (Room)	644,590	634,235	621,666	1,390,325	1,417,410	1,455,876
Rural and township health centers	35,762	34,943	33,917	1,390,325	1,417,410	1,455,876
rural clinic	608,828	599,292	587,749		-	-
5. Other institutions	3,000	3,299	3,738	24,067	34,540	29,021
Total	1,022,922	1,030,935	1,032,918	9,100,700	9,450,110	9,749,933

Data source: China Health Commission’s 2020–2022 China Health Care Development Statistical Bulletin (38–40).

**Figure 1:**
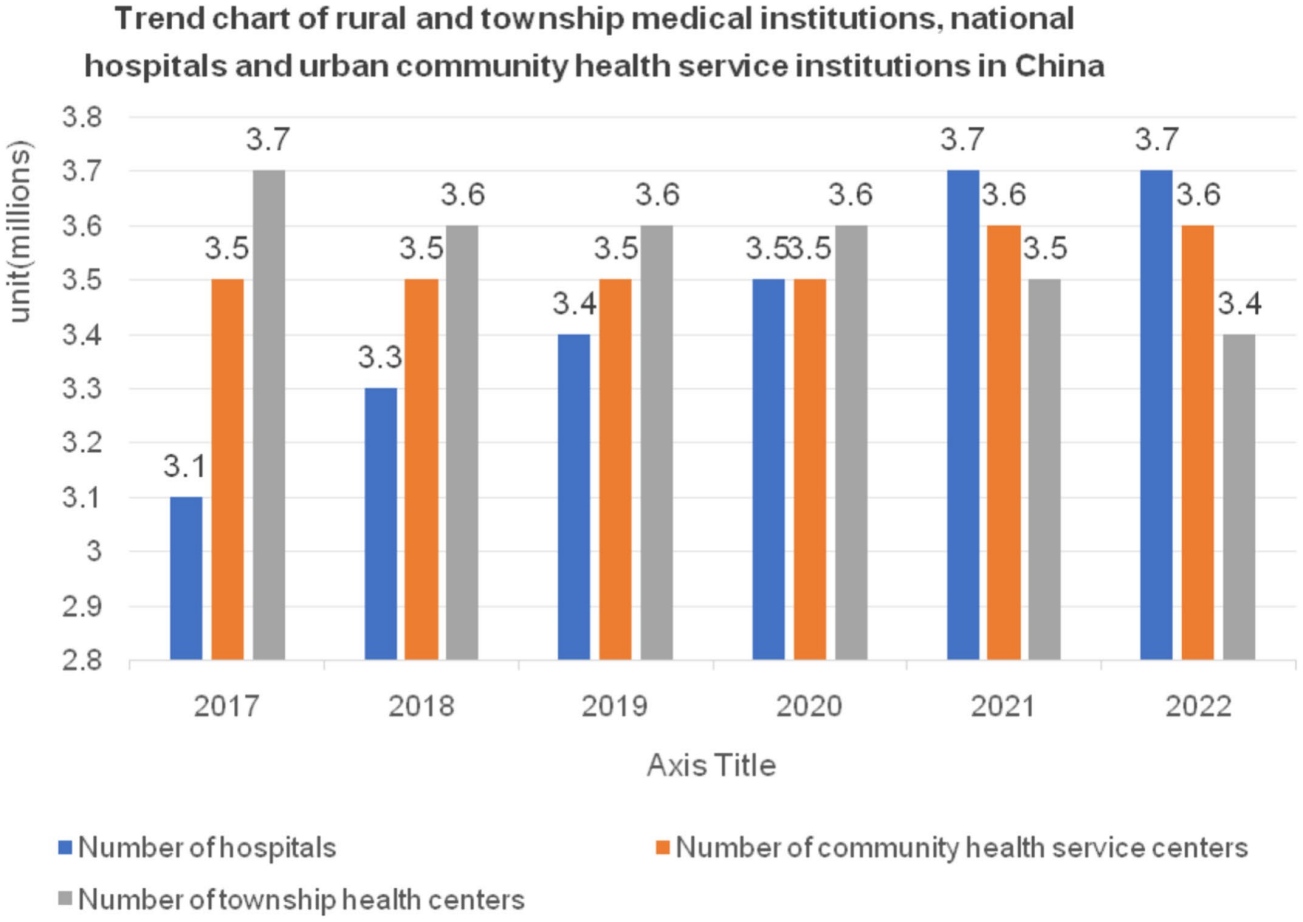
Change trends of rural and township medical institutions, national hospitals, and urban community health service institutions in China. Data source: China Health Commission’s 2022 China Health Care Development Statistical Bulletin (40).

Figure 1 is a chart of the change trends of China’s rural township health centers national hospitals and urban community health service institutions from 2020 to 2022. The total number of hospitals in China remained consistent at 35,000, 37,000, and 37,000, respectively. For urban community-based healthcare service institutions, the numbers were 33,000, 34,000, and 36,000 over the same period. Rural township health clinics saw figures of 36,000, 35,000, and 34,000 during the 3 years. One major factor contributing to the decline in rural township hospitals is the ongoing urbanization process in China. As a result, fewer people live in rural areas, which in turn reduces the need for these hospitals. Patients in rural locations sometimes have limited access to treatment beds, few amenities, and as few as one or two healthcare providers at each village health center. Due to a lack of individual analysis, these centers frequently offer healthcare services that are inadequate or nonexistent.

**Figure 2:**
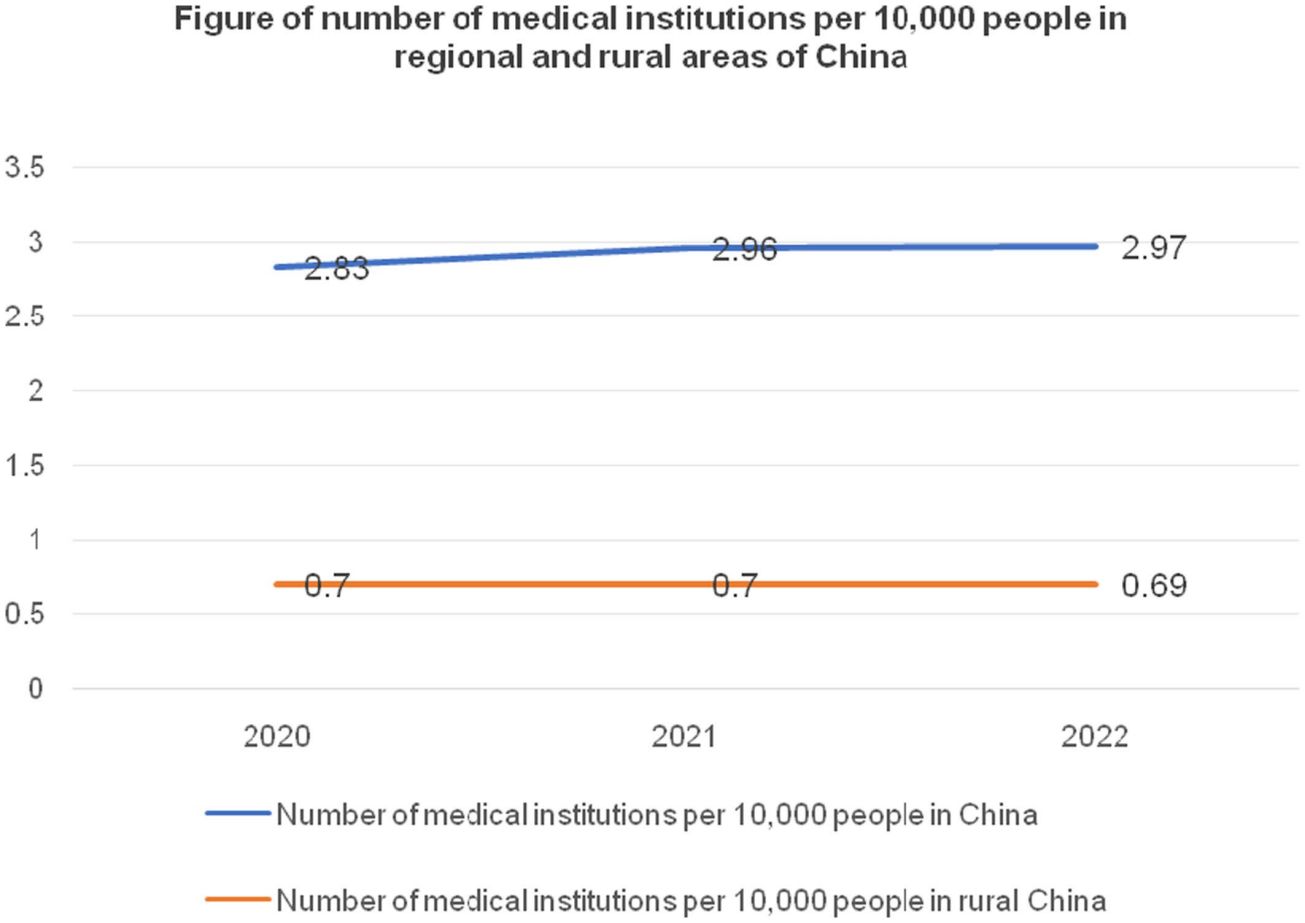
Number of medical institutions per 10,000 people nationwide and in rural areas from 2020 to 2022. Data source: China Health Commission’s 2020–2022 China Health Care Development Statistical Bulletin (38–40).

**Figure 3:**
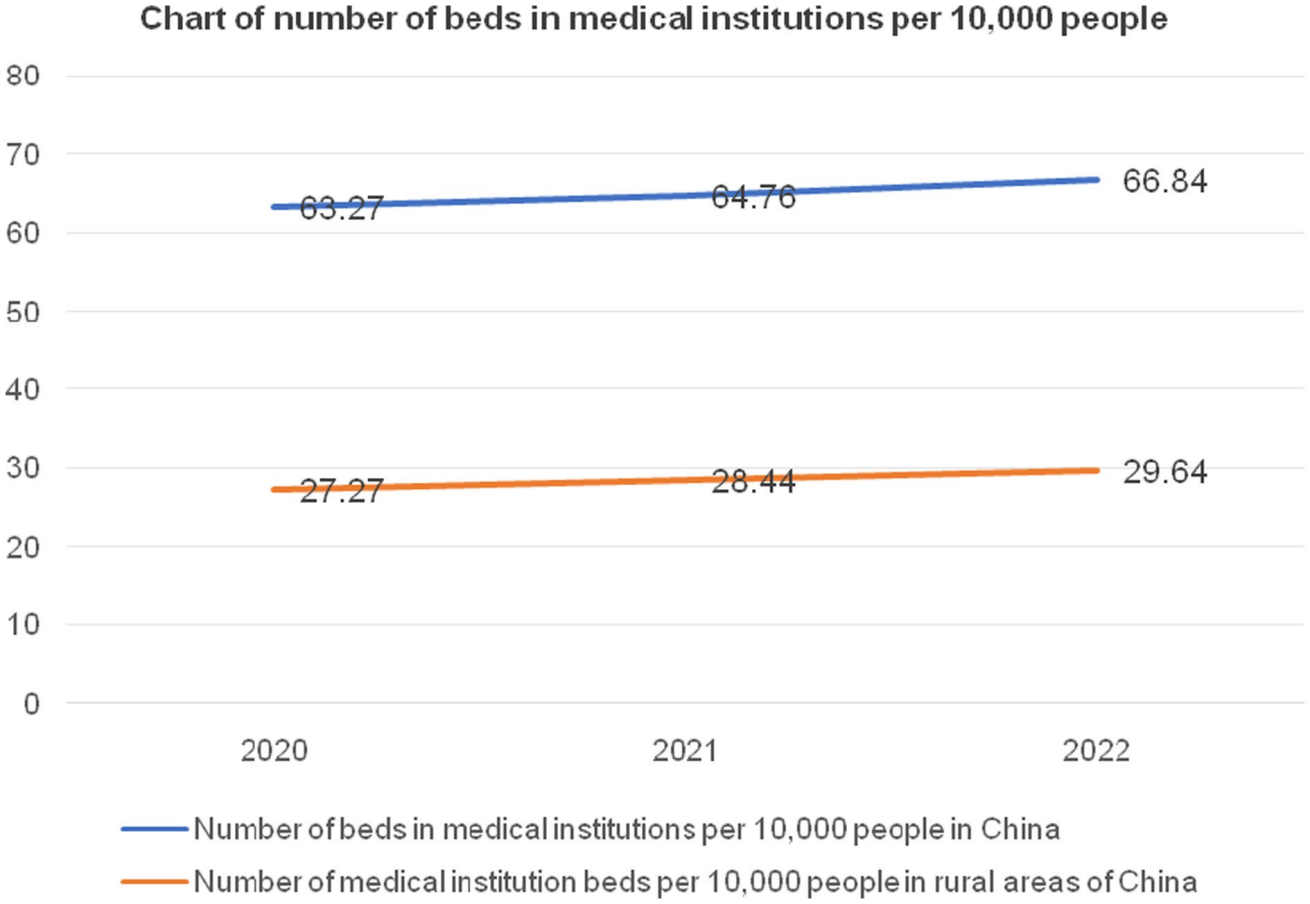
Number of beds in medical institutions per 10,000 people nationwide and in rural areas from 2020 to 2022. Data source: China Health Commission’s 2020–2022 China Health Care Development Statistical Bulletin (38–40).

From the trend depicted in Figure 1, the number of township health clinics has been consistently experiencing a gradual decline, while the quantity of hospitals and community health institutions in urban areas has shown a slow but steady upward trend. It is necessary to take population fluctuations into account when analyzing the capacity of rural healthcare institutions and beds to meet developmental needs (41). The following statistical study of healthcare facilities on a national and rural level accounts for population changes. This analysis does not cover public health supervision and healthcare institutions because of their unique focus on the prevention, supervision, and care of residents’ health, which sets them apart from healthcare institutions that mainly offer general treatment. In rural areas, village health centers often only have one or two healthcare workers on staff, no treatment beds, and very minimal facilities. As a result, the healthcare services they provide are often inadequate (42). Therefore, village-level health centers are excluded from this analysis. According to the data in Table 1, from 2020 to 2022, and excluding specialized public health supervision and healthcare institutions and village health centers, the total number of moderately-sized healthcare institutions in the country was 399,602, 418,367, and 432,733, respectively. Over the 3 years, the total bed capacity of healthcare institutions nationwide was 8,804,637, 9,148,544, and 9,436,375, respectively. From 2020 to 2022, the number of township health clinics in rural areas was 35,762, 34,943, and 33,917, respectively. Over the same period, the bed capacity of rural township health clinics was 1,390,325, 1,417,410, and 1,455,876, respectively. From 2020 to 2022, the total population of mainland China was 1,411.77 million, 1,412.60 million, and 1,411.75 million, respectively. The rural population during the same period was 509.78 million, 498.35 million, and 491.04 million, respectively. Figures 2, 3 show the national and rural healthcare institution-to-population ratios and bed capacity per 10,000 people, respectively. As shown in Figure 2, the national healthcare institution-to-population ratio in 2020 was 2.83 (399,602/141,177 million people), with a rural area ratio of 0.7 (35,762/50,978 million people). Figure 3 shows that in 2020, there were 62.37 beds in healthcare institutions for every 10,000 persons in the United States (8,804,637/141,177 million people) and 27.27 beds in rural areas (1,390,325/50,978 million people). For both 2021 and 2022, comparable computations were performed.

### 4.2 Investigation of health technical personnel

In 2022, the total number of health technical personnel nationwide reached 11.658 million, an increase of 414,000 compared to 2021 and 980,000 compared to 2020. Both hospitals and community-based healthcare institutions experienced varying degrees of growth in health technical personnel. As of the end of 2022, rural healthcare institutions in China employed 1.991 million health technical personnel, representing an increase of 9,000 compared to 2021 and a decrease of 82,000 compared to 2020 (38–40). Examining the trend in the changes in health technical personnel, there is an inverse relationship between the change in rural health technical personnel and the national trend. This correlation can be attributed to the development dynamics in rural areas and overall changes in the population (Table 3).

**Table 3:** Number of health technicians in various medical institutions across the country from 2020 to 2022.

Institutions category	Number of health technicians		
	2020 年	2021 年	2022 年
1. Hospital	677.5	711.5	735.3
Among them: public hospitals	529.2	552.7	571.7
Private hospital	148.2	158.9	163.6
2. Community primary medical and health institutions	184.7	201.7	212.4
Among them: community health service center	44.4	47.6	50.6
Community health service station	11.4	11.6	11.7
Medical nursing station, etc.	128.9	142.5	150.1
3. Rural health and medical institutions	127,7	128.5	132.6
Among them: rural and township health centers	127.7	128.5	132.6
4. Professional public health prevention and supervision agencies	72.7	76.4	78
Where: Centers for Disease Control and Prevention	14.5	15.8	16.9
Maternal and child health care institutions, etc.	51.8	54.9	55.6
Health Supervision Institute (Center)	6.4	5.7	5.5
5. Other institutions	5.2	6.3	7.5
total	1067.8	1124.4	1165.8

Unit: 10,000 people. Data source: China Health Commission’s 2020–2022 China Health Care Development Statistical Bulletin (38–40).

**Figure 4:**
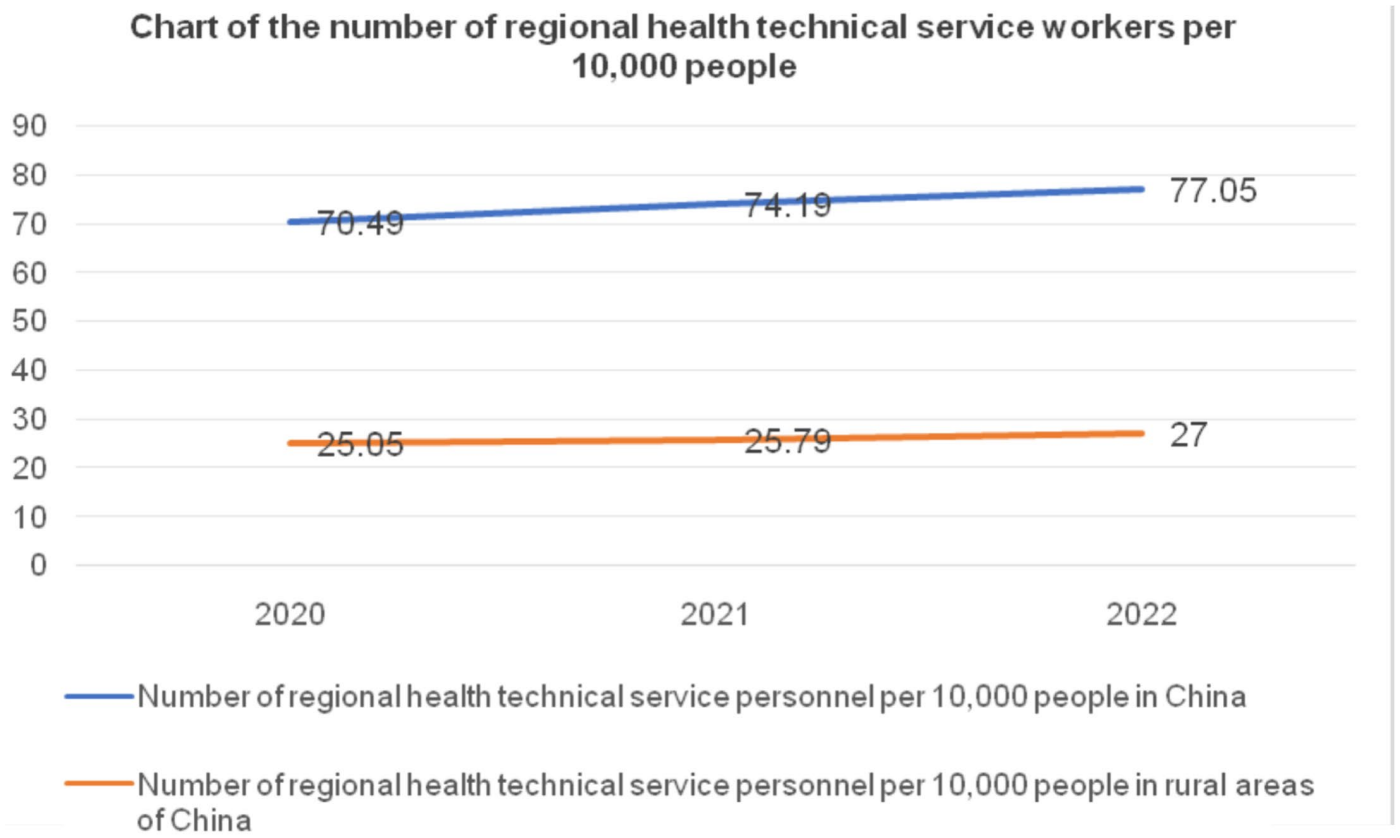
Number of health technicians per 10,000 people nationwide and in rural areas from 2020 to 2022. Data source: China Health Commission’s 2020–2022 China Health Care Development Statistical Bulletin (38–40).

To conduct a detailed analysis of the changes in rural health technical personnel, a comparison comparative assessment was undertaken between the ratio of health technical personnel per 10,000 individuals in rural regions and the national average. This assessment aimed to evaluate the disparities and deficiencies in the number of health technical personnel in rural areas due to the distinct focus of professional public health prevention and supervision institutions on residents’ health prevention, supervision, and healthcare, differing from general treatment-oriented healthcare institutions, this analysis excluded health technical personnel from professional public health prevention and supervision institutions for uniformity in comparison. From 2020 to 2022, the number of health technical personnel in rural China was 1.277, 1.285, and 1.326 million, respectively. After excluding public health prevention and supervision personnel, the national total for health technical personnel over these 3 years was 9.951, 10.48, and 10.878 million, respectively. By Considering the total population of mainland China from 2020 to 2022 (1411.77, 1412.60, and 1411.75 million) and the rural population (509.78, 498.35, and 491.04 million), the ratio of health technical personnel per 10,000 people for both the nation and rural areas can be seen in Figure 4. In Figure 4, the number of health technical personnel per 10,000 people nationwide in 2020 was 70.49 (995.1/141.177 million people), while in rural areas, it was 25.05 (1.277/50.978 million people). The same methodology was applied for the years 2021 and 2022.

Health technical personnel include practicing physicians, nurses, pharmacists, and other categories, with practicing physicians being the most crucial as they directly interact with patients. According to Article 7 of the “Management Measures for the Registration of Physicians,” both practicing physicians and assistant practicing physicians can practice within the administrative division of medical, preventive, and healthcare institutions. However, practicing physicians have a broader range of practice locations. Practicing physicians can practice within the provincial-level administrative division where the medical institution is located, while assistant practicing physicians are limited to the county-level (district) administrative division where the medical institution is located and cannot practice across different locations. As per the “Interim Measures for the Qualification Examination for Medical Practitioners,” practicing physicians and practicing (assistant) physicians need to pass the medical qualification examination to obtain their qualifications. Practicing physicians or assistant practicing physicians must hold a “Physician Qualification Certificate” (43). In rural township health centers, there is a significant proportion of practicing (assistant) physicians. Table 4 provides statistics data on practicing physicians and assistant practicing physicians in national and rural township health institutions.

**Table 4:** Statistics of practicing (assistant) physicians nationwide and in rural areas.

Statistical indicators	2020	2021	2022
Number of practicing physicians and practicing (assistant) physicians nationwide	408.6	428.8	443.5
Number of practicing physicians and practicing (assistant) physicians in rural towns and village health institutions	98.5	100.1	103.9
Number of practicing physicians (including assistants) per 10,000 people nationwide	28.94	30.35	31.41
Number of practicing (assistant) physicians per 10,000 people in rural areas	19.32	20.09	21.16

Unit: 10,000 people. Data source: China Health Commission’s 2020–2022 China Health Care Development Statistical Bulletin (38–40).

**Figure 5:**
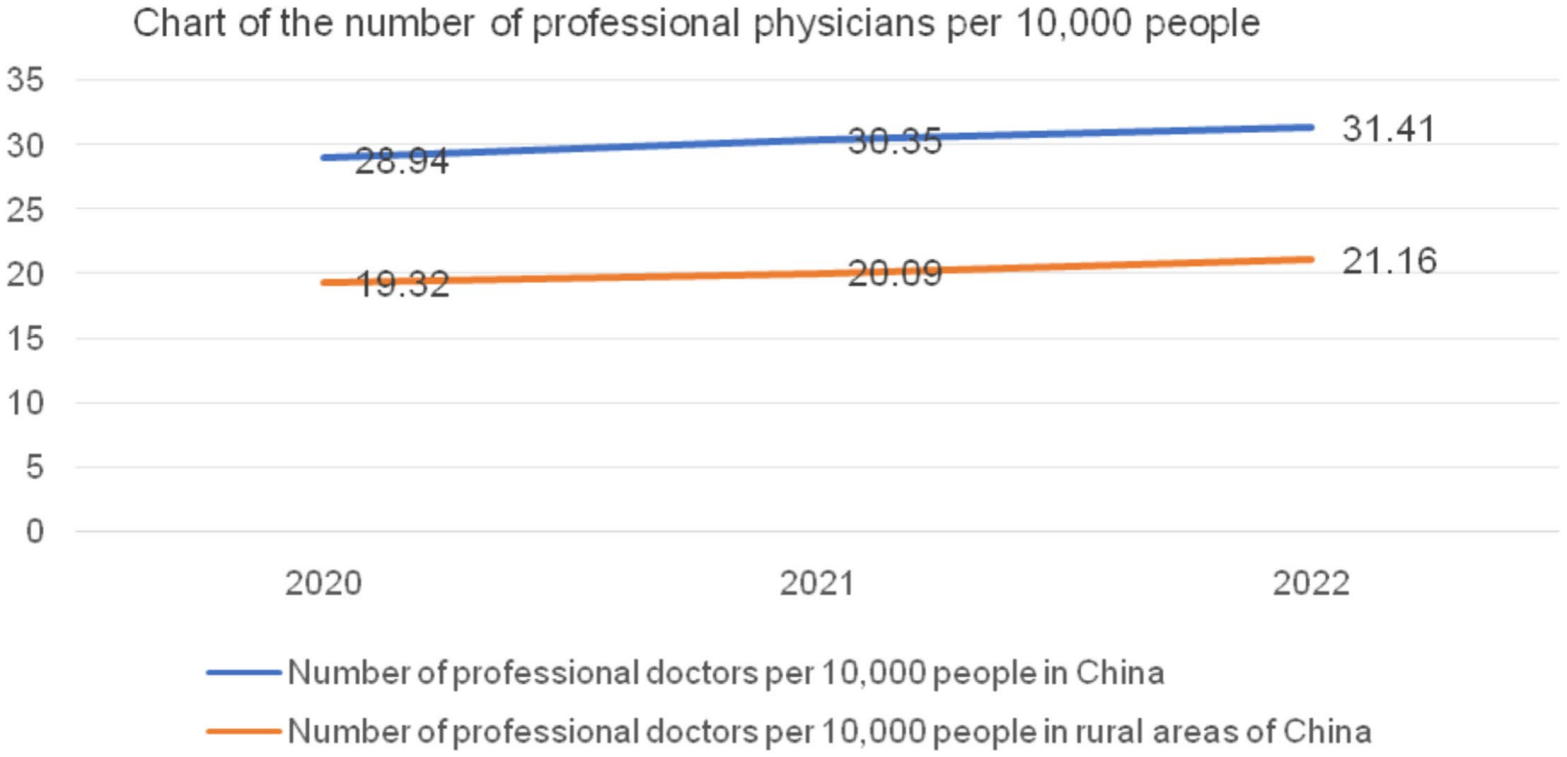
Number of practicing physicians (including assistants) per 10,000 people nationwide and in rural areas from 2020 to 2022. Data source: China Health Commission’s 2020–2022 China Health Care Development Statistical Bulletin (38–40).

The calculation of the number of practicing physicians (including assistant physicians) per 10,000 people nationwide in Table 3 is as follows: In 2020, it was 28.94 = the total number of practicing physicians and (assistant) physicians in the country (4.086 million people)/the total national population in 2020 (1411.77 million people). The calculation of the number of practicing physicians (including assistant physicians) per 10,000 people in rural areas is as follows: In 2020, it was 19.32 = the total number of practicing physicians and (assistant) physicians in rural areas (52 + 46.5 million people)/the total rural population in 2020 (509.78 million people). The calculation of the number of physicians per 10,000 people in rural areas includes two levels: township health centers and village-level health institutions. The trends in the changes in the number of physicians per 10,000 people nationwide and in rural areas are illustrated in Figure 5.

### 4.3 Investigation into the medical service situation of rural health institutions

Rural health institutions provide medical services, including the diagnosis and treatment of common illnesses and minor injuries, emergency care for acute diseases and accidental injuries, management of chronic diseases, health check-ups, and other services. The delivery of medical services by rural township health institutions is typically influenced by diverse factors such as regional characteristics, the national healthcare system, and economic conditions (44).

This research project conducted a survey and analysis of the medical services offered by rural health institutions in China, with a focal emphasis on parameters including the count of practicing physicians and assistant physicians in rural township health centers, total outpatient consultations, administration to inpatients care at, I rural township health centers, daily patient load per physician, and daily bed occupancy per physician. Subsequently, these datasets were then compared with the national average to assess the level of medical services provided by rural township health institutions.

Table 5 summarizes and presents these statistics.

**Table 5:** Medical services in rural health and medical institutions from 2020 to 2022.

Statistical indicators	2020	2021	2022
Number of beds in township health centers (10,000)	139	141.7	145.6
Number of practicing physicians and practicing (assistant) physicians in rural and township health centers (10,000 people)	52	52.5	53.7
Number of practicing physicians and practicing (assistant) physicians in rural village-level health branches (offices) (10,000 people)	46.5	47.6	50.2
Rural diagnosis and treatment visits (100 million visits)	25.3	25	24.9
Number of admissions to rural and township health centers (10,000 persons)	3383.3	3,223	3,239
The average number of diagnoses and treatments performed by doctors at the township and village levels in rural areas every day	10.2	9.95	9.55
The average number of inpatient beds occupied by doctors in township health centers per day	1.35	1.3	1.27
Hospital bed utilization rate (%)	50.4	48.2	46.9
The average length of stay for discharged patients (days)	6.6	6.6	6.5

Data source: China Health Commission’s 2020–2022 China Health Care Development Statistical Bulletin (38–40).

According to the stipulations and calculation rules based on key statistical indicators from the National Bureau of Statistics of China, the interpretation and calculation rules for the relevant indicators in Table 5 are as follows: Total outpatient visits refer to the overall number of medical consultations, including outpatient visits, emergency visits, consultations provided, and medical consultations by the staff of the health center.

This also encompasses individual health check-ups conducted outside the center and health counseling sessions. Physicians’ average daily patient load is calculated as the total number of outpatient visits divided by the average number of physicians and further divided by 251. Here, 251 represents the average annual working days in a year, considering a deduction for holidays and rest days.

Taking the actual number of occupied bed days, multiplying it by the hospital’s service days (often taken as 365 days), dividing it by the average number of physicians, and then dividing it by 365 is the formula for physicians’ average daily bed occupancy. A patient’s average length of stay in the hospital is calculated as the number of discharges divided by the total number of bed days occupied by inpatients from admission to discharge. The number of hospital beds, number of practicing physicians and assistant physicians, number of outpatient visits, number of inpatient admissions, bed utilization rate, average length of hospital stay, and other statistics are taken from the National Health Commission of China’s Annual Statistical Bulletin on the Development of Health and Health Care, which is where Tables 4, 5 are located.

In Table 5, the calculation for the daily patient load per physician in rural towns and village-level healthcare institutions in 2020 is 10.2, derived as the total outpatient visits (2.53 billion visits) divided by the total number of physicians (52,000 + 46,500) in town hospitals and village-level health institutions, and further divided by 251 days. The calculations for physician daily patient load in 2021 and 2022 in rural areas are performed using the same method. In Table 4, the calculation for the daily bed occupancy per physician in town hospitals in 2020 is 1.35. This is calculated using the actual occupied bed days (139 million bed days) multiplied by the bed utilization rate (50.4%), then multiplied by 365 days, and subsequently divided by the total number of physicians (52,000) in town hospitals, this result further divided by 365 days. As physicians, village-level health institutions in rural areas typically have small scales and no beds for inpatient admission, the physicians in these institutions are not included in the calculation for daily bed occupancy. The calculations for physician daily bed occupancy in 2021 and 2022 in town hospitals are performed using the same method. In Table 5, the calculations for the national average daily patient load per physician and the national average daily bed occupancy per physician follow the same method as described in Table 4.

This methodology that relies on data analysis offers a strong foundation of evidence to create specific policies and initiatives that aim to tackle inequalities in the distribution of healthcare resources in rural areas.

## 5 Results

Despite continuous government investments in rural healthcare resources, China’s healthcare service system still faces issues of unreasonable distribution and configuration of medical resources. The differences seen in the medical resources between the national average and rural areas reflect the significant divide between urban and rural areas in China. The primary cause for this disparity is the “siphon effect” that occurs when large urban hospitals drain the health resources of smaller, community-based medical facilities. Healthcare is an essential kind of human capital. When it is distributed improperly, it worsens the development disparity between rural and urban regions. This misallocation also contributes to systemic problems such as shortages of medical resources and inefficiencies.

The data presented in Table 6 illustrates the differences between rural areas and the national average concerning medical institutions, bed numbers, and other aspects.

**Table 6:** Medical services in national health and medical institutions from 2020 to 2022.

Statistical indicators	2020	2021	2022
Number of beds in medical institutions nationwide (10,000)	880.46	914.85	943.64
Practicing physicians and (assistant) physicians	408.6	428.8	443.5
Number of diagnosis and treatment visits (100 million)	77.4	84.7	84.2
Number of hospital admissions (10,000 people)	23,013	24,726	24,686
Average number of doctor visits per day	7.55	7.87	7.56
Physicians’ average daily charge of inpatient beds	1.56	1.59	1.51
Hospital bed utilization rate (%)	72.50	74.6	71
The average length of stay for discharged patients (days)	8.5	9.2	9.2

Data source: China Health Commission’s 2020–2022 China Health Care Development Statistical Bulletin (38–40).

In Table 6, the disparity is calculated by subtracting the rural figures from the national figures, with the number of medical institutions per 10,000 people excluding specialized public health supervision and health care institutions, as well as village-level health clinics without beds in rural areas. From Table 7, notable differences are evident between the average quantities in rural areas and the national average in terms of healthcare institutions, bed numbers, health technical personnel, and specialized practicing physicians (including assistants). The average per 10,000 people in rural areas is notably lower than the national average.

**Table 7:** Differences in health and medical resources between the country and rural areas.

Statistical indicators		2020	2021	2022
Number of medical institutions per 10,000 people	Nationwide rural area difference	2.83	2.96	2.97
		0.7	0.7	0.69
		2.13	2.26	2.28
Number of beds in medical institutions per 10,000 people	Nationwide rural area difference	62.37	64.76	66.84
		27.27	28.44	29.64
		35.1	36.32	37.2
Number of health technicians per 10,000 people	Nationwide rural area difference	70.49	74.19	77.05
		25.05	25.79	27
		45.44	48.4	50.05
Number of practicing physicians (including assistants) per 10,000 people	Nationwide rural area difference	28.94	30.35	31.41
		19.32	20.09	21.16
		9.62	10.26	10.25

Data source: China National Health Commission’s 2020–2022 China Health Care Development Statistical Bulletin and the above chart calculations.

According to the statistics provided in Table 5, spanning from 2020 to 2022, the average daily number of patient diagnoses and treatments conducted by rural township and village-level practicing and assistant physicians were 10.2, 9.95, and 9.55, respectively.

The average daily number of diagnoses and treatments performed by practicing and assistant physicians nationwide were 7.55., 7.87, 7.56 times, the three-year average number of diagnoses and treatment visits in rural areas is 2.24 times more than the national number [(10.2 + 9.95 + 9.55)/3 − (7.55 + 7.87 + 7.56)/3]; Physician Days of Rural Township Health Centers from 2020 to 2022 The average number of inpatient bed days charged by doctors across the country were 1.35, 1.3, and 1.27, respectively. The average number of inpatient bed days charged by doctors nationwide were 1.56, 1.59, and 1.51. The three-year average number of inpatient bed days charged by doctors in rural areas was 0.25 bed days less than the national average; from 2020 to 2022, the average length of stay for patients hospitalized and discharged from rural township health centers will be 6.6, 6.6, and 6.5 days, respectively. The average length of stay for patients hospitalized and discharged from hospitals and medical institutions across the country will be 8.5, 9.2, and 9.2 days, respectively. For rural patients hospitalized the average number of days per year is 2.4 days less than the national average. The data shows that the number of diagnoses and treatments burdened by rural doctors is greater than the national average, and the pressure on doctors to receive medical treatment is greater than the national average; the average length of stay for rural patients is less than the national average, and the average number of hospitalization beds per day burdened by rural doctors is higher than the national three-year average in rural areas. It is less, indicating that the service conditions for patient treatment in rural medical institutions are lower than the national average, and the medical conditions and accommodation levels of hospitals and other medical institutions outside rural areas are better than those in rural township health centers.

## 6 Discussion

This study investigates and analyzes the resource status of primary healthcare institutions in rural China, providing insights into medical facilities, technical personnel, and healthcare services, comparing them with the national average to study the deficiencies and disparities in rural healthcare resources. The findings of this study align with prior research that has demonstrated the disparity in healthcare resources between urban and rural areas in China. Public health emergencies provide distinct problems to various circumstances, such as pregnant women, impacting their physiological, psychological, and social well-being. This study specifically examines the circumstances behind the COVID-19 pandemic in China (45). In reality, the Chinese government has consistently rolled out a series of policies and measures to narrow the gap between rural and national healthcare resources. These measures include increasing investment in rural healthcare, improving the mechanism for training medical personnel, and promoting telemedicine. As early as 2009, China’s Ministry of Health, Ministry of Finance, and National Population and Family Planning Commission jointly issued the “Opinions on Promoting the Gradual Equalization of Basic Public Health Services,” with the objection of achieving comprehensive coverage of national basic public health service projects and significantly reduce the gap in public health services between urban and rural areas. The document envisioned that by 2020, the mechanism for the gradual equalization of basic public health services would be substantially refined, major diseases and major health risk factors effectively controlled, and the health status of both urban and rural residents further improved. The primary cause of the disparities observed in various rural healthcare resources compared to the national average is the urban–rural gap. As of the end of 2022, China’s rural population amounted to 491.04 million, individuals, constituting 34.78% of the total national population of 1,411.75 million (excluding Hong Kong, Macao, and Taiwan the urban population comprises 65.22%, of the total yet hospitals and the majority of public health resources are concentrated in large and medium-sized cities, resulting in differences between national and rural healthcare resources). There has been a gradual decline in the number of township health centers, while the count of hospitals and community healthcare institutions in cities has been consistently increasing. Similarly, the study (46) uncovered a very minor discrepancy between various regions and different tiers of hospitals in China, while certain areas still necessitate enhancement. There is a serious shortage of licensed medical professionals in township hospitals, which forces some facilities to hire unqualified healthcare technicians. Only the director of some township hospitals is qualified to practice medicine as an assistant, but other doctors may acquire rural doctor certifications (47). It is not uncommon for rural township hospitals to have a severe lack of practicing assistant physicians, whereas village health clinics rarely have any on staff. In rural areas, healthcare facilities are struggling to meet the medical treatment demands of their patients due to a lack of healthcare technical staff and practicing assistant physicians qualifying as (48). The primary factor contributing to the deficit of healthcare technical personnel is the relatively higher concentration of medical institutions in urban areas, which attract more healthcare professionals. Certain rural areas face challenges of healthcare professional outflow, as doctors and nurses prefer working in urban healthcare settings. This contributes to the shortage of medical personnel in rural areas.

The government needs to develop epidemic or pandemic strategies using data and customize them for certain demographic groups to control the pandemic condition (49). In terms of healthcare institutions and facilities, Chinese hospitals are typically organized into various levels, categorized into first, second, third, and fourth grades (special grade). The classification is primarily determined by the number of beds: fewer than 100 beds, such as township health centers, are considered first-grade hospitals; between 100 and 500 beds, with over 100 beds, are classified as second-grade; over 500 beds are designated as third-grade. Besides these three grades, there is also a fourth grade (special grade) reserved for exceptionally large hospitals. Additionally, hospitals are classified into A, B, and C levels based on technical expertise, medical conditions, and management standards. First-level rural township hospitals do not have the A, B, and C classifications because their technical proficiency and medical conditions do not meet the requirements for these classifications. Second and third-grade hospitals may have A, B, and C classifications. According to statistical data, on the one hand, there are approximately 0.7 healthcare institutions with beds per 10,000 people in rural China, contrasting with around 2.9 healthcare institutions with beds per 10,000 people nationwide. Rural areas fall short of the national average by approximately 2.2 healthcare institutions with beds per 10,000 people. Concerning the bed count in healthcare institutions, rural areas exhibit a shortfall of about 36 fewer beds per 10,000 people compared to the national average. On the other hand, based on technical proficiency, medical conditions, and management standards, rural township hospitals are all considered first-grade hospitals and do not have the A, B, or C classifications that denote comprehensive service hospitals The healthcare conditions in rural areas are rudimentary and cannot handle patients with complex medical conditions. Whether in terms of the quantity of healthcare institutions and facilities or the level of medical technology, rural healthcare resources cannot directly meet the demands of rural residents for medical institutions. A comparison of rural areas to the rest of the country over the last 3 years reveals that there are around 48 fewer healthcare technical personnel per 10,000 people and about 10 fewer practicing (including assistant) physicians per 10,000 people than the national average. According to a study, the digital economy negatively impacts the effectiveness of public health services mainly in two ways: by encouraging the use of social media and by increasing the disparity in healthcare access between urban and rural areas. Furthermore, these effects and methods of transmission display spatial variability (50). In terms of healthcare services in rural healthcare institutions, between 2020 to 2022, the rural average frequency of several medical consultations per person was 2.24 times higher than the national average. Similarly, a prior study investigated the disparities in access to and requirements for general medical care based on the level of rurality among adult inhabitants of Washington State. The study analyzed several obstacles to healthcare access across rural and urban areas, revealing notable disparities in barriers at the system level but not at the individual level. After accounting for the characteristics of the respondents (51), data indicates that rural physicians manage a greater volume of medical consultations per person compared to the national average. Since secondary, tertiary, and special-grade hospitals are concentrated in large and medium-sized cities, rural patients with complex conditions generally seek treatment directly at these urban hospitals (52). With the migration of rural patients to urban areas, the average frequency of medical consultations per person for rural physicians is 2.24 times higher than the national average, suggesting that rural physicians have a relatively larger patient load. From another perspective, this implies a shortage of rural physicians. The average length of hospitalization for rural patients is less than the national average, and the daily average hospital bed days per rural physician are lower over the 3 years compared to the national average. This indicates that the medical conditions in rural township hospitals are below the national average (53). The underlying reason is the imbalance in medical resources and facilities. In urban areas especially large and medium-sized cities, medical facilities are relatively advanced, with high-level hospitals and clinics (54). However, in some rural areas, due to economic constraints, medical facilities may be relatively rudimentary with lower equipment levels. Due to the disparity in medical resources, urban residents typically have access to higher-quality medical services, while rural residents may face issues of inconsistent medical service quality (55).

This study is unique because it conducts a thorough examination of medical facilities, technical people, and healthcare services in rural China. It offers a comprehensive view of the resources available in primary healthcare institutions. The analysis identifies the precise areas where discrepancies exist and need to be addressed by comparing these features with national averages. Although this study offers a thorough examination of resource availability in primary healthcare institutions in rural China, it is crucial to recognize specific constraints. The analysis predominantly depends on statistical data obtained from National Health Commission bulletins, which restricts the scope to quantitative parameters. By integrating qualitative data sources, such as conducting field surveys or interviews with healthcare personnel and patients, a more profound understanding of the actual experiences and difficulties encountered in rural healthcare settings can be obtained (56). In addition, the study’s emphasis on national-level patterns may mask regional discrepancies or inequalities within rural areas across various provinces or municipalities. Furthermore, the study does not thoroughly investigate the socioeconomic, policy, or geographic aspects that contribute to the observed differences in healthcare resources between urban and rural areas. Although there are limits, the findings emphasize important gaps and deficits in healthcare resources in rural areas. This underscores the necessity for ongoing efforts to overcome these inequities and guarantee fair access to high-quality healthcare services for rural residents in China (57).

## 7 Conclusion

The study’s findings emphasize the notable discrepancies and inadequacies in the resource condition of primary healthcare institutions in rural China when compared to the national average. The current deficiencies in medical facilities, healthcare technical people, and the quality of healthcare services in rural areas highlight the immediate necessity for focused measures to address the disparity in healthcare resources between urban and rural communities. To tackle these difficulties, it is imperative to adopt a comprehensive strategy that includes augmenting funding for rural healthcare infrastructure, establishing incentive programs and retention initiatives for healthcare practitioners, advocating for telemedicine and remote consultation, enhancing the skills of rural healthcare workers, and fortifying the referral system between primary care facilities and higher-level hospitals. To provide equitable access to quality healthcare services for both urban and rural populations in China, authorities can address these concerns by implementing evidence-based policies and practices. This study offers significant insights and establishes a starting point for monitoring progress and evaluating the effectiveness of programs designed to diminish gaps in healthcare resources between urban and rural areas. It is imperative to recognize that guaranteeing access to high-quality healthcare is not solely a matter of distributing resources, but also a problem of social equity and long-term viability. Allocating resources to enhance rural healthcare infrastructure and staff will positively impact the overall welfare and efficiency of rural communities, ultimately yielding benefits for the entire nation. To tackle the intricate issues encountered by rural healthcare systems in China, it is imperative to have persistent endeavors, cooperation among diverse stakeholders, and sustained dedication in the long run. By giving priority to enhancing healthcare resources in rural areas, the government may make a substantial stride toward attaining universal health coverage and fostering health equity for all its residents.

## Data availability statement

The original contributions presented in the study are included in the article/supplementary material, further inquiries can be directed to the corresponding author.

## Author contributions

ZZ: Writing – review & editing, Writing – original draft, Visualization, Validation, Resources, Investigation, Data curation, Conceptualization.
